# Exploring Social Impairment in Those with Opioid Use Disorder: Linking Impulsivity, Childhood Trauma, and the Prefrontal Cortex

**DOI:** 10.21203/rs.3.rs-4202009/v1

**Published:** 2024-04-08

**Authors:** Thais Arruda, Laura Sinko, Paul Regier, Altona Tufanoglu, Adrian Curtin, Anne Teitelman, Hasan Ayaz, Peter Cronholm, Anna Rose Childress

**Affiliations:** Temple University; Temple University; University of Pennsylvania; University of Pennsylvania; Drexel University; University of Pennsylvania; Drexel University; University of Pennsylvania; University of Pennsylvania

**Keywords:** Opioid Use Disorder, social functioning, impulsivity, childhood trauma, prefrontal cortex, brain, behavior

## Abstract

**Background:**

Challenges with social functioning, which is a hallmark of opioid use disorder (OUD), are a drawback in treatment adherence and maintenance. Yet, little research has explored the underlying mechanisms of this impairment. Impulsivity, a known risk factor for OUD, and corresponding neural alterations may be at the center of this issue. Childhood adversity, which has been linked to both impulsivity and poorer treatment outcomes, could also affect this relationship. This study aims to understand the relationship between impulsivity and social functioning in those recovering from OUD. Differences in the prefrontal cortex will be analyzed, as well as potential moderating effects of childhood trauma.

**Methods:**

Participants with (N = 16) and without (N = 19) social impairment completed a survey (e.g., social functioning, Barrat’s Impulsivity Scale, Adverse Childhood Experiences (ACEs) and cognitive tasks while undergoing neuroimaging. Functional near infrared spectroscopy (fNIRS), a modern, portable, wearable and low-cost neuroimaging technology, was used to measure prefrontal cortex activity during a behavioral inhibition task (Go/No-Go task).

**Results:**

Those who social functioning survey scores indicated social impairment (n = 16) scored significantly higher on impulsivity scale (t(33)= −3.4, p < 0.01) and reported more depressive symptoms (t(33) = −2.8, p < 0.01) than those reporting no social impairment (n = 19). Social functioning was negatively correlated with impulsivity (r=−0.7, p < 0.001), such that increased impulsivity corresponded to decreased social functioning. Childhood trauma emerged as a moderator of this relationship, but only when controlling for the effects of depression, B=−0.11, p = 0.023. Although both groups had comparable Go/No-Go task performance, the socially impaired group displayed greater activation in the dorsolateral (F(1,100.8) = 7.89, p < 0.01), ventrolateral (F(1,88.8) = 7.33, p < 0.01), and ventromedial (F(1,95.6) = 7.56, p < 0.01) prefrontal cortex during impulse control.

**Conclusion:**

In addition to being more impulsive, individuals with social impairment exhibited differential activation in the prefrontal cortex when controlling responses. Furthermore, the impact of impulsivity on social functioning varies depending on ACEs demonstrating that it must be considered in treatment approaches. These findings have implications for addressing social needs and impulsivity of those in recovery, highlighting the importance of a more personalized, integrative, and trauma-informed approach to intervention.

## Background

### Opioid use disorder (OUD)

Opioid use disorder (OUD) is a significant public health issue characterized by the compulsive consumption of opioids, which are substances (such as heroin, morphine, codeine, and fentanyl) associated with pain relief. Worldwide, there were approximately 600,000 deaths related to drug use in 2019, with almost 80% of them associated with opioids(1). In the United States, there has been a drastic increase in overdoses since 2019, coinciding with the COVID-19 pandemic, which resulted in nearly 110,000 reported overdose deaths by the end of 2022 (1), largely driven by opioids (~ 72%). This recent surge in overdoses calls for an increase in supporting recovery and improving treatment. Studies have shown that patient-provider relationships and support networks are fundamental in treatment adherence, maintenance, and satisfaction (2). However, patients in recovery often exhibit challenges in developing and maintaining these relationships, which can represent a drawback. To provide more effective care, it is fundamental to understand the underlying mechanisms behind the social challenges that patients in recovery might face.

### Social functioning

Social functioning is defined as the ability to participate in social roles and activities, and it is often affected by the chronic use of opioids (3, 4). Challenges with or related to social functioning are key criteria in the diagnosis of OUD (DSM-5). Social functioning encompasses many skills, such as empathy, communication, adaptability, and self-regulation. These allow individuals to navigate interpersonal interactions, detect social cues, and ultimately develop healthy relationships. The Social Cognitive Theory posits that individuals acquire and develop these attributes through observation and role models (5). In his *Social Cognitive Theory of Self-regulation,* Bandura examines the relevance of self-regulatory mechanisms, underscoring the capacity to control one’s emotions, thoughts, and behaviors as key during social interactions. In line with this idea, previous work has suggested that inhibitory control might be essential to social functioning (6). If controlling impulses is crucial to successfully participating in society, impulsivity may be central to OUD patients’ social challenges.

### Opioid use disorder (OUD) and self-regulation.

One of the critical factors associated with OUD is impulsivity. Individuals who are impulsive exhibit higher risk-taking behaviors (7, 8), seeking pleasure and reward while overlooking possible consequences. Impulsivity exacerbates drug-seeking behaviors, which may lead to the development of OUD. Indeed, previous studies have found that impulsivity is a risk factor for opioid misuse as well as the development of OUD (9, 10). From a neurocognitive perspective, impulsivity is often associated with the term ‘disinhibition,’ referring to the top-down control of more reflexive (i.e., subcortical, or bottom-up) responses (11). The prefrontal cortex (PFC), an area often associated with impulse control and inhibition of responses, is known to be affected by the chronic use of opioids. Studies have shown that OUD is associated with disrupted activity in the PFC (12, 13), which may lead to impairments in inhibitory control. In heroin-dependent patients, a study found that impulsive behavior was correlated with a decrease in gray matter volume in the PFC (14). Notably, research has consistently shown differential activation of the PFC for heroin users compared to non-users during inhibition tasks (15, 16). Hence, impulsivity may contribute to the development and maintenance of OUD, while OUD can lead to alterations in the structure and function of the PFC, exacerbating impulsive and drug-use behaviors.

Despite the extensive amount of literature supporting impulsivity as a risk factor for OUD, there are few studies investigating how it further impacts individuals during recovery. Lack of impulse control in social interactions, disruption of social rules, and inability to regulate emotional responses can all hinder one’s ability to interact socially. Hence, self-regulation represents a critical factor in the control of social behavior (17). Consistently, on the neural level, lesions to the PFC have been associated with social impairment (18, 19, 20). Moreover, disruptions in the PFC circuitry have been observed in patients with psychiatric disorders commonly associated with deficits in social functioning, such as schizophrenia and autism spectrum disorder (21). Therefore, the underlying dysregulation of PFC circuitry may affect one’s ability to self-regulate, leading to challenges in social situations. Overall, underlying mechanisms in the PFC may contribute to impulsive behaviors that impact social functioning during treatment.

### Adverse Childhood Experiences.

Exposure to adversity during childhood, including physical, sexual, and emotional abuse, as well as neglect, has also been linked to OUD. Among individuals diagnosed with OUD, approximately 41% of women and 16% of men are estimated to have a history of childhood sexual abuse (22). Since childhood is a pivotal time in development, experiencing trauma during this period can have long-lasting effects that encompass physical health, behaviors, and neurobiology. For example, studies have shown that childhood adversity is linked to both functional and structural alterations in the brain (23). On a behavioral level, those with a history of childhood trauma are more likely to seek drugs as a coping mechanism to deal with the emotional burden (24). Adversity during childhood has also been associated with both impulse control (25, 26) and interpersonal (27) challenges in adulthood. For those with OUD, in particular, childhood trauma has been linked to poorer treatment outcomes (22). This connection may be explained by the heightened social challenges faced by those with comorbid OUD and a history of childhood trauma. Social functioning may be an underexplored, yet promising, area to improve outcomes for those with OUD (28, 29). However, it remains unexplored whether adverse childhood experiences impact the relationship between impulsivity and social functioning in individuals recovering from OUD.

**This study** sought to understand what lies behind the social challenges patients face in recovery. First, it investigated if those with impaired social functioning are more impulsive. Then, it explored if the PFC activation during impulse control was altered for those with poor social functioning. Finally, it analyzed whether childhood trauma moderates these relationships at a behavioral level. We hypothesized that those with social impairments would be more impulsive and display significantly more activity in the dorsolateral PFC during a novel version of the Go-NoGo task (probes impulse control with affective images - Puppies Go! Spiders No!). We further hypothesized that childhood trauma moderated the relationship between impulsivity and social functioning, where those with greater traumatic experiences displayed more impulsivity and consequently increased challenges in social functioning. Understanding how previous experiences may shape behavior during recovery can help tailor interventions to improve treatment adherence. These intricate and highly individualized experiences may be a gateway to maximize chances of recovery and later social reintegration.

## Methods

This is a secondary analysis using data from two pilot studies, examining the impact of opioid use and sexual violence on executive functioning. Thirty-five participants (women, n = 29) were recruited for this study. The present study analyzed the survey, neuroimaging, and behavioral task data.

### Recruitment

Individuals were recruited through community organizations that support those on a medication-assisted treatment for OUD in a large metropolitan area, using flyers, email listservs, and presentations at in-person programmatic events. Interested people reached out through phone or email to be screened for our study. Participants were eligible to participate in the study if they were (1) between the ages of 18–60, (2) on a medication-assisted treatment for OUD, (3) able to attend an in-person brain imaging session, (4) not pregnant, and (5) not presenting with a severe mental health condition (e.g., schizophrenia). All participants offered informed consent before participation, and the University of Pennsylvania Institutional Review Board approved all procedures in this study.

### Procedure

The study was divided into two visits. During the first visit, which lasted around 2 hours, participants completed an online survey and the Penn Computerized Neurocognitive Battery (30) tasks. They then received a resource list and were asked to schedule a second two-hour visit. In the second visit, participants performed another series of cognitive tasks while connected to a non-invasive brain monitoring device called functional near-infrared spectroscopy (see section on *fNIRs)*. Individuals were compensated for their time.

### Survey Measures

#### Demographics

Demographics included age, zip code, gender, race, ethnicity, and socioeconomic measures such as the highest education level, mother’s education level, current employment, and income.

#### Social functioning

Social functioning was assessed using the PROMIS Ability to Participate in Social Roles and Activities (31). This study used the short form with eight questions evaluating the perceived ability to participate in social activities (e.g., “I have trouble doing all of my regular leisure activities with others”). Items were reversed scored, so higher scores represented fewer perceived limitations and, hence, higher social functioning. Using the PROMIS scoring system (32), the final scale score was transformed into a t-score with a mean of 50 and a standard deviation 10. The t-score metric allows us to compare the participants’ scores to the US general population, with scores below one standard deviation of the mean representing some impairment. T-scores are further subdivided into mild (45 – 40), moderate (40 – 30), and severe (below 30) impairment. We used both a summed score (continuous variable) and a categorical variable for analysis purposes. Those with social functioning levels one standard deviation below the general population average (equivalent to a t-score of 40) were categorized as part of the socially impaired (SI) group. At the same time, those with scores of 40 or above were included in the not socially impaired (N-SI) group.

#### Impulsivity

Impulsivity was assessed with Baratt’s Impulsiveness Scale (BIS) (33), a self-report measure composed of thirty questions (e.g., “I do things without thinking”). It uses a 4-item metric that ranges from “rarely/never” to “almost always/always,” with higher scores representing greater impulsivity. The BIS has been previously used and validated in different populations, with an alpha coefficient of around 0.69 to 0.83 (34)

#### Adverse Childhood Experiences

Adverse Childhood Experiences were measured using the Philadelphia Adverse Childhood Experiences (PHL ACEs) survey (35). This is an expanded version of the conventional Adverse Childhood Experiences (ACE) containing 16 items used to assess exposure to abuse and trauma during childhood. This version is especially useful for certain sociodemographic groups, including people of color and low-income backgrounds, as it incorporates questions sensitive to those groups’ experiences (35). The expanded version includes potential community-level adversity, assessing exposure to racism, bullying, and neighborhood violence. Following the conventional ACEs scoring sheet, a total sum score was generated, with greater scores representing greater exposure to childhood adversity. Although previous studies have revealed that significant health outcomes begin to appear with scores of 4 or higher (36), this approach disregards the variances and nuances that might arise from exposure to higher levels of adversity. Considering the increased levels of childhood trauma observed in our sample (see description in the Results section), we chose to use a cumulative score approach to encompass these variations. This method has been previously used and replicated in many studies (37).

#### Depression

Depression was assessed using two different measures: the Quick Inventory of Depressive Symptomology (QIDS) and the PROMIS Item Bank v. 1.0 - Emotional Distress - Depression. The QIDS is a 16-item scale used to measure depressive signs and symptoms, with a focus on emotional distress (38). Each question was scored on a 0–3 severity scale, and the total score was calculated by adding up all the responses in a range of 0 to 48 - higher scores meaning higher depressive symptoms. The QIDS has been demonstrated to be both reliable (a = 0.80 to 0.94) (39) and strongly correlated to other validated measures such as the Beck Depression Inventory and the Hamilton Rating Scale for Depression (39). The PROMIS Depression scale (40) is a 28-item scale scored on a 1–5 (“never” to “always”) range with more outstanding scores meaning greater display of symptoms. It has been proven to be both valid and reliable as a tool to assess depression symptoms across different samples (41).

### Neuroimaging Data Collection

#### Functional near-infrared spectroscopy (fNIRS)

Functional near-infrared spectroscopy (fNIRS) is a wearable neurotechnology used to measure changes in cortical oxygenation changes using near infrared light with non-invasive wearable sensors over the scalp (42).Traditionally, research involving populations in recovery or with a history of trauma exposure has often relied on conventional devices such as functional magnetic resonance imaging (fMRI). In this study, we chose to use fNIRS for three main reasons: portability, affordability, and comfort. Contrary to conventional technologies, fNIRS is a small and lightweight device, allowing the data collection to be carried out in a variety of environments such as clinics, hospitals, and the field (43, 44), which reduces location restrictions and increase accessibility for participants. Following the size discrepancy, fNIRS also has a lower cost when compared to other traditional devices (44), reducing the cost of the overall research process.

Moreover, in contrast to the discomfort posed by fMRI, fNIRS has a simplified head-band structure, which prevents participants from undergoing long periods of stillness in confinement and enduring loud noises. This may help reduce patients’ anxiety and distress, minimize potential triggers, and increase comfort during data collection (43–45). Hence, fNIRS emerges as a technique that is sensitive to the unique needs and challenges of individuals with exposure to trauma and in recovery from opioid use. Aside from being affordable, this is a more trauma-informed and community-based technology, helping foster a more accommodating research environment and making it ideal for our study.

For neural data collection, participants sat in a room with a computer and a wearable fNIRS sensor Model 1200 (fNIR Devices LLC) system with a flat sensor pad placed over the anterior PFC and secured with elastic fabric. While completing a task (described below), activity in the PFC was measured using four light sources and ten detectors resulting in 16 optodes (cortical measurement areas) as described before (46). There are two light wavelengths (730 and 850 nm) with a temporal resolution of 500 milliseconds. The arrangement of the light source and the detectors on the device resulted in 16 active optodes, or channels (47) distributed from dorsal to ventral and lateral to medial brain.

#### The fNIRS task

**Figure 1:**
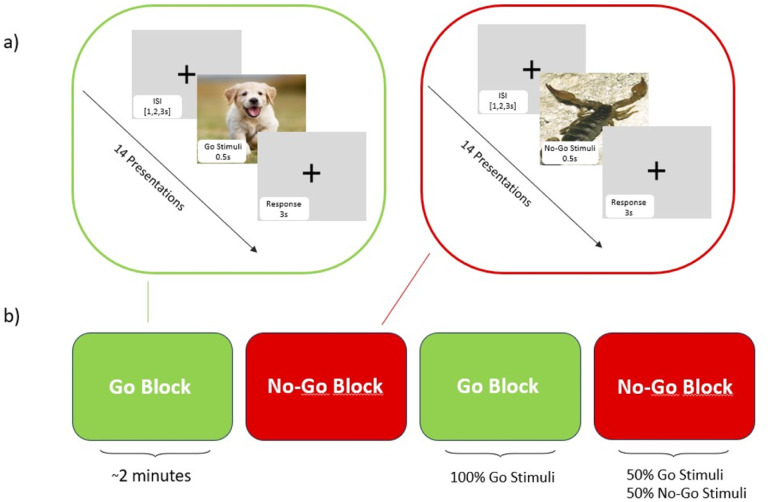
Affective Go/No-Go task. ***(a)*** Each block presents individual stimuli: puppies (Go) or a combination of puppies and scorpions (No-Go). Participants must respond within 3 seconds, and this is followed by a variable interstimulus interval (ISI) of 1, 2, or 3 seconds. ***(b)***The trial consists of alternating ‘Go’ and ‘No-Go’ blocks with specified proportions of stimuli: two ‘Go’ blocks contain only puppy images (100% Go stimuli), and two ‘No-Go’ blocks feature an equal mix of puppy and scorpion images (50% Go and 50% No-Go stimuli).

The fNIRS task used was an affective version of the well-known Go-No-Go task, also called the Spiders-No, Puppies-Go task (48) (see Fig. 1) on a computer with a 15.6-inch monitor using PsychoPy^®^ software (49). This affective task uses appealing (puppies) and aversive (scorpions) images to increase the ecological validity of the traditional task. Instead of the abstract stimuli typically presented (i.e., letters and numbers), using evocative images makes the task more engaging (48). This is particularly important in this study as we use it to observe neural activation; hence, approach/avoidance stimuli can help simulate responses more similar to environmental conditions.

As in the typical task, participants received instructions and completed practice trials before commencing the task. They were instructed to press the space bar when shown ‘Go’ stimuli and refrain from pressing when shown ‘No-Go’ stimuli. The task consisted of four blocks, comprising two ‘Go’ and two ‘No-Go’ conditions, in which subjects were shown stimuli for 0.5s and had 3 seconds to respond. This was followed by an inter-stimulus interval (ISI) - fixation cross appearing on the screen - for a random interval ranging from 1, 2 or 3 seconds. Each block consisted of 14 images, with an inter-block interval of approximately 10 seconds. The Go trial is used as a form of control, as only appealing photos are shown (100% Go stimuli), while in the No-Go trial, both aversive (50%) and appealing (50%) images are shown, enabling the assessment of impulse control abilities.

### Analysis:

#### Signal processing.

For each participant, raw light intensity data (wavelengths of 730nm and 850nm) were continuously sampled from 16 anterior PFC regions at a rate of 2 Hz. The preprocessing pipeline included low-pass filtering (FIR with a linear phase) at 0.1 Hz to eliminate high-frequency noise and physiological artifacts such as cardiac and respiration cycles. fNIRS data for each block were extracted using time synchronization markers received through a serial port during the experiment. Subsequently, the acquired data was analyzed to calculate alterations in oxygenated hemoglobin (HbO) concentrations using the Modified Beer-Lambert Law. Motion artifacts were corrected by applying Temporal Derivative Distribution Repair as described by Fishburn et al. (2019) (50). The hemodynamic response at each optode was averaged across time for each trial block to provide a mean hemodynamic response at each optode for each block. Relative changes in oxyhemoglobin (HbO) and deoxyhemoglobin (HbR) for each activation condition were calculated relative to distinct local baselines measured during the first ten samples at the beginning of the Go and NoGo blocks. Changes in oxyhemoglobin and deoxyhemoglobin for the two activation conditions were calculated relative to respective local baseline segments.

#### Statistical Analyses

Statistical Analyses were conducted using IBM SPSS Statistics (Version 27) and RStudio. The significance criterion was set to *a* = 0.05. We used descriptive statistics, including mean and standard deviations, to describe sociodemographic characteristics. To understand group differences (socially impaired versus not) of depression (QIDS, PROMIS-Depression) and impulsivity (BIS) scores, two-sample t-tests were conducted. We also used Pearson’s correlations to examine potential linear relationships between impulsivity (BIS) and social functioning (total [PROMIS-Social Functioning] score). To explore possible moderation effects of childhood trauma, we fitted a linear model (estimated using ordinary least squares) to predict this interaction. Variables significantly related to our dependent or independent variables were included as covariates in our model to control for confounds.

Considering depression is both commonly comorbid with OUD (51) as well as a consistent outcome of childhood trauma experiences (52), it seemed crucial to control for the potential effects of this variable. In line with previous literature (53), individuals in our sample with social functioning challenges displayed greater levels of depressive symptoms. Therefore, we decided to look beyond the depression symptom burden by controlling for potential variations in our moderation analysis.

In addition, we analyzed the Affective Go/No-Go task at both behavioral and brain activation levels. Analysis of variances (ANOVAs) was used to compare group performance differences, examining response time and accuracy (correct responses per trial). We used linear mixed effects model analysis for neuroimaging data to estimate the main effects of group (socially impaired vs. not) vs. conditions (Go vs. No-Go trials) and their interaction. This model was chosen because it handles repeated measures more effectively than ANOVAs. Our task presented repeated data points for the same trial type, and using this analysis enabled the data within each group to be treated as related instead of independent. The dependent variable was HbO (used as a proxy for brain activation) at each optode, from one to sixteen. In this context of multiple comparisons, False Discovery Rate (FDR) corrections were used to control for the inflation of positive results.

## Results

### Sample

Thirty-five men and women were recruited for this study. Of those, 32 self-described as having an addiction or substance use disorder and were on medication-assisted treatment for OUD, and 29 shared that they used opioids in the past year. According to the PROMIS Ability to Participate in Social Roles and Activities t-score system, 16 participants had some social impairment, ranging from mild (n = 8) to moderate and severe (n = 8). Participants experienced a considerable degree of adversity during childhood, with only two individuals reporting no exposure to adverse childhood experiences (ACEs). The overall sample displayed an average cumulative score of 5.89 on the PHL ACES scale. Breaking it down into quartiles, 7 participants reported 1–3 ACEs, 13 reported 4–6, 6 reported 7–8, and 7 reported over eight adverse experiences. For more information, see Table 1.

### Survey Results

#### Is social impairment associated with higher impulsivity scores?

Impulsivity and social functioning were negatively correlated, such that increased impulsivity was associated with decreased social functioning (r=−0.7, p< 0.001). T-tests also revealed that the socially impaired group had significantly higher impulsivity scores (M = 80.2) than the group without social impairment (M = 67.3, t(33)= −3.4, p< 0.01).

#### Do those with social impairment differ in depression levels compared to those without?

The socially impaired group reported significantly higher depressive symptom scores (M = 19.3) compared to those without (M = 12.9, t(33) = −2.8, p < 0.01). This was true for both depressive symptom instruments used in this study: QIDS (t(33) = −2.8, p = 0.009) and PROMIS (t (28)= −2.4, p = 0.02).

Does childhood trauma moderate the relationship between impulsivity and social functioning when controlling for depression symptoms?

**Figure 2:**
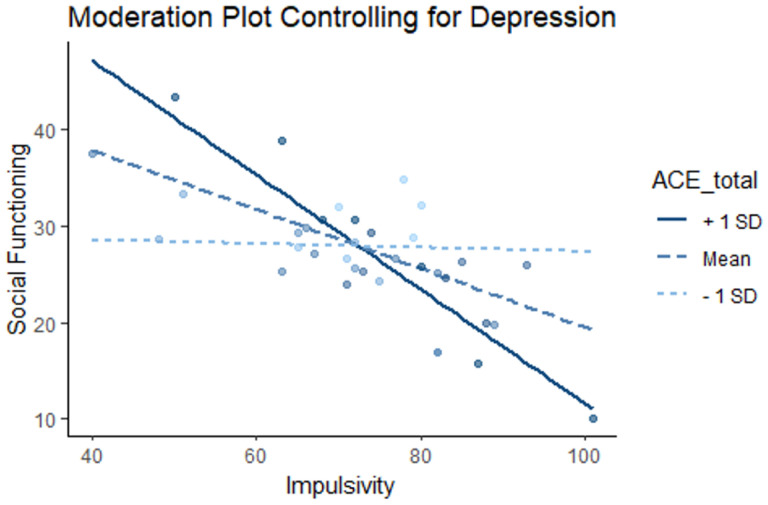
Moderation plot for ACEs, impulsivity, and social functioning, while controlling for depression symptoms. The solid line represents ACEs score one standard deviation above the mean the dotted blue line represents ACEs score at the mean and the light blue dotted line represents scores one standard deviation below the mean. There is a significant moderator effect of ACEs on the relationship between impulsivity and social functioning (B=−0.11, p=0.023). Simple slopes analysis revealed that the relationship between social functioning and impulsivity was not significant for individuals with low ACEs (light blue dotted line) (b = −0.02, SE = 0.18, p = 0.92), but significant for those with high ACEs (solid bue line) (b = −0.59, SE = 0.16, p < 0.001).

We investigated whether the relationship between social functioning and impulsivity is moderated by exposure to childhood adversity, including depressive symptoms (using the QIDS) as a covariate. This yielded a significant moderator effect of ACEs on the relationship between impulsivity and social functioning (B=−0.11, p = 0.023). Additional simple slopes analysis revealed that, when looking at low ACES (one standard deviation below the mean), the relationship between social functioning and impulsivity was not significant (b = −0.02, SE = 0.18, p = 0.92). Conversely, when looking at high ACES (one standard deviation above the mean), the relationship was significant (b=−0.59, SE = 0.16, p < 0.001). This interaction remained significant for those with experiences of childhood adversity close to the mean (M = 5.11, B=−0.31, SE = 0.13, p < 0.05; See Fig. 2).

The solid line represents ACEs score one standard deviation above the mean the dotted blue line represents ACEs score at the mean and the light blue dotted line represents scores one standard deviation below the mean. There is a significant moderator effect of ACEs on the relationship between impulsivity and social functioning (B=−0.11, p = 0.023). Simple slopes analysis revealed that the relationship between social functioning and impulsivity was not significant for individuals with low ACEs (light blue dotted line) (b = −0.02, SE=0.18, p = 0.92), but significant for those with high ACEs (solid bue line) (b = −0.59, SE= 0.16, p< 0.001).

### Neuroimaging Results

Are there differences in brain activation between the groups (socially impaired vs. not) when engaging in impulse control tasks?

Using the Go-No-Go task, a well-known and established task to measure abilities to control impulses, we examined performance brain activity in the PFC between those with and without social impairment.

**Figure 3:**
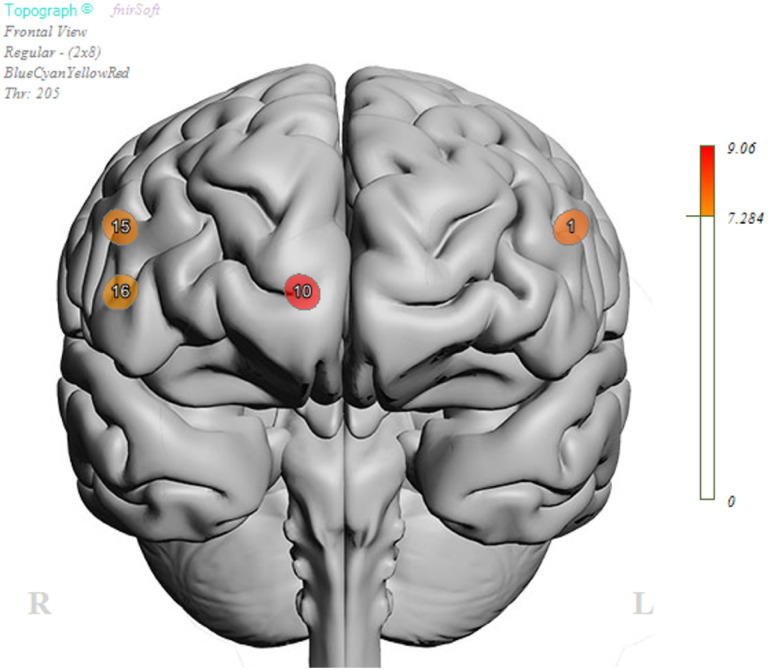
Parametric plot for main effects of Group (SI vs. N-SI) during affective Go-NoGo task. This parametric plot shows results from a linear mixed effects model, with corresponding F-score values representing significant (FDR-corrected) main effects of group at optode 1 (left dorsolateral PFC), optode 10 (right ventromedial PFC), optode 15 (right dorsolateral PFC), and optode 16 (right ventrolateral PFC).

**Figure 4:**
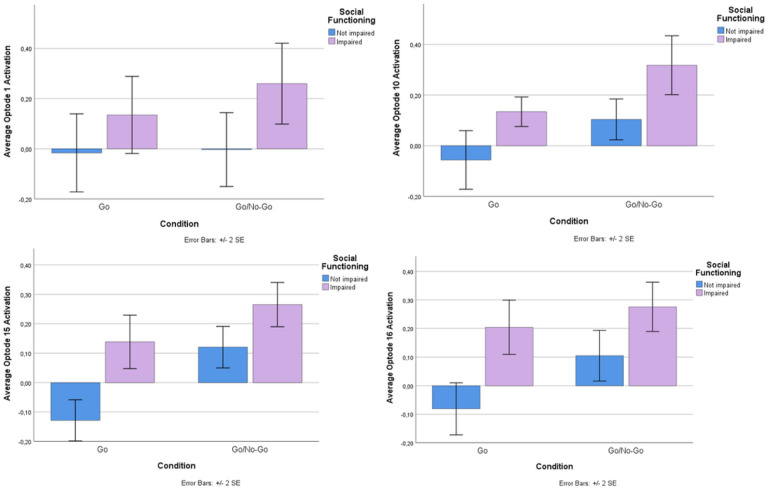
Bar graphs for average HbO activity from fNIRS Optodes that had a significant main effect of group (SI vs SI-N). For each individual barplot y-axis indicates average HbO changes in each optode. Blue bars represent the SI-N group while purple represent the SI group. On the left of each figure, HbO activity is shown for Go condition and on the right activity is shown for Go/No-Go. Whiskers represent the standard error of the mean (SEM).

ANOVA analysis revealed no differences in performance, neither in response time (F(25, 1) = 0.013, p = 0.911) nor in accuracy of responses (F(25, 1) = 0.652, p = 0.427). Despite equal performance on the task, linear mixed model analysis revealed activation differences at the level of the brain (Table 2). There was a main effect of group (FDR corrected) in Optode 1 (F(1,100.8) = 7.89, p < 0.01), Optode 10 (F(1,75.8) = 9.06, p < 0.01), Optode 15 (F(1,95.6) = 7.56, p < 0.01), and Optode 16 (F(1,88.8) = 7.33, p < 0.01) (see Fig. 3). Estimated fixed effects suggest that the socially impaired group had an increased (primarily dorsolateral) cortical response during the task compared to the non-socially impaired group (see Fig. 4). The main effects of condition (Go vs. No-Go) were found only in Optode 10 (F(1,75.8) = 6.58, P = 0.012) and Optode 15 (F(1,95.6) = 6.23, p = 0.014). No interaction effect was observed between the condition and group.

This parametric plot shows results from a linear mixed effects model, with corresponding F-score values representing significant (FDR-corrected) main effects of group at optode 1 (left dorsolateral PFC), optode 10 (right ventromedial PFC), optode 15 (right dorsolateral PFC), and optode 16 (right ventrolateral PFC).

For each individual barplot, y-axis indicates average HbO changes in each optode. Blue bars represent the Sl-N group while purple represent the SI group. On the left of each figure, HbO activity is shown for Go condition and on the right activity is shown for Go/No-Go. Whiskers represent the standard error of the mean (SEM).

Exploratory: Are there differences in brain activation between the groups (socially impaired vs. not) during impulse control tasks when controlling for depression?

Considering the previously stated prevalence of depression symptoms in this population, we decided to further control for depression in the neural analysis portion of our study. Results revealed that only two Optodes of interest remained significant when performing the same linear mixed models analysis with depression as a covariate: Optode 1 (F(1,97.9) = 13.54, p < 0.001), and Optode 10 (F(1,75.32) = 7.42, p < 0.01), while the main effects of social impairment on Optode 15 (F(1,96.2) = 3.47, p = 0.066) and Optode 16 (F(1,87.8) = 3.60, p = 0.062) did not reach significance after controlling for depression symptoms. Previously observed main effects of task condition (Go vs. No-Go) remained significant in Optode 10 (F(1,74.9) = 6.47, p = 0.013) and in Optode 15 (F(1.,95.7) = 6.22, p = 0.014).

## Discussion

Identifying underlying mechanisms of social functioning challenges in OUD is critical for developing more effective interventions. Our study found that patients in recovery from OUD who exhibit some level of social impairment have higher impulsivity scores, greater depressive symptoms, and increased PFC activity when attempting to regulate their impulses compared to those without any detectable social impairment. We also found that, when controlling for depressive symptoms, ACEs moderated the relationship of social impairment and impulsivity, such that those with a higher degree of trauma exposure displayed increased impulsivity and decreased social functioning. These results highlight the importance of integrative care framed in trauma informed approaches, taking into account not only mental health symptom burden but also trauma history to help promote social functioning, which facilitates the ability to build and form relationships critical for recovery.

Previous literature has found that impulsivity is both a risk factor and a contributor to the maintenance of OUD (54), with some highlighting that more impulsivity is linked to poorer treatment outcomes (55). However, very few studies investigate what lies behind this self-regulation challenge and how it further impacts patients during recovery. One study traced negative indicators of social functioning in patients recovering from OUD (56), analyzing demographic (age, gender, race, etc.) as well as socioeconomic (employment and residential status) factors. Yet, the study did not explore potential behavioral variables such as impulsivity. Inhibition is thought to be central to controlling social behavior (17). This has been corroborated in prior studies such as the one authored by Von Hippel & Gonsalkorale (2005), which revealed that cognitive inhibition predicted more appropriate social behavior (6). Our study extends this idea to those in recovery, as increased impulsivity is linked to social impairment. Therefore, beyond being a risk factor and a contributor to OUD, impulsivity also further impacts patients’ relationships. Since patient-provider relationships and support networks (57) are crucial during treatment, impulsivity represents a drawback in recovery.

This study adds to this initial finding, exploring the potential mechanisms that underlie this relationship. The added layer of trauma might impact the impulsivity of those in recovery (58). Peck et al. (2022) found that those with comorbid Post-Traumatic Stress Disorder (PTSD) and OUD were more impulsive in the context of negative emotions compared to those in recovery without PTSD (58). Furthermore, research has pointed out that experiencing adversity during childhood is linked to poor social outcomes (59) and impulsivity (60, 61). Aligned with these ideas, our study found that, when controlling for depressive symptoms, the relationship between impulsivity and social functioning is moderated by childhood trauma history. To our knowledge, this was the first study to find the moderation role of childhood adversity in the context of social functioning and impulsivity for those in recovery. This result suggests that the relationships between trauma, impulsivity, and OUD might be additive.

It is not surprising that those with social impairment revealed more significant levels of depression symptoms. Previous studies have found that, in those who have SUD, social impairment is more prominent when it is comorbid with mental health issues (62, 63). Considering its relevance in the context of OUD, depressive symptoms were considered at every step of our analysis to explore potential relationships that exist beyond its notorious effects. As discussed, childhood adversity emerged as a moderator only when controlling the effects of depression symptomology. This may indicate that trauma history affects patients above and beyond the mental health burden alone. Although unexpected, this finding aligns with previous literature that uncovered the broader impact of trauma history on survivors, surpassing mental health concerns (64).

In this study, we explored brain activity during an affective inhibitory task to better understand the neural underpinnings of social functioning. In particular, we aimed to understand if those with social impairment had differential brain activity while engaging in impulse control. While the socially impaired group exhibited higher levels of self-reported impulsivity, there were no differences of performance on the fNIRS Go-No-go task. The lack thereof is not unique to this study; similar results have been reported in other studies employing this paradigm (65–67). This result could be attributed to the task’s inherent simplicity, corresponding to the observed ceiling effect on performance. Conversely, this facilitated the identification of notable differences in brain activation during comparable behavior.

The SI group revealed greater activation in the dorsolateral (Optodes 1 and 15), ventrolateral (Optode 16), and ventromedial (Optode 10) PFC compared to the N-SI group. In line with existing literature (68), differences in neural activation during response inhibition were primarily found in the lateral regions of the PFC. Experiments with clinical populations that exhibit impulse control challenges, such as Attention-Deficit/Hyperactivity Disorder (ADHD) and mania, also revealed activation differences in the ventrolateral (69, 70) and dorsolateral (67) PFC during the Go-No/Go task. The studies, however, reported attenuated activation of these regions for the clinical groups compared to control during impulse inhibition, while in our sample, the socially impaired group displayed increased activation. One of the primary differences between this study and the others is the affective nature of our Go/No-Go task, which may elicit higher responses in those with impairment.

The implication of both right (Optode 1) and left (Optode 15) dorsolateral PFC aligns with its established role in top-down regulation of behavior, especially in task-relevant responses (71). Notably, research has pointed out that directly stimulating this region can improve response inhibition (72, 73). Hence, it would be reasonable to assume that detected heightened activation corresponds to enhanced inhibition. In our sample, however, the enhanced activation of the dorsolateral PFC did not align with increased performance. One possible explanation for this lies in the suggested role of the dorsolateral PFC in cognitive flexibility. It posits that increased activation of this region is associated with adaptive cognitive control (74, 75), helping implement adjustments to adapt to errors or conflicts detected. This could represent a compensatory mechanism, as Weissman et al. (2008) proposed that the role of the dorsolateral PFC in adaptive cognitive control extends to social contexts (76). Individuals with social impairment may require greater mental effort to adapt their behavior, inhibit responses, and adjust to social norms. This might provide a hint for resilience pathways: those with social impairment may have learned how to be resilient to underlying neurological differences - as revealed by the equivalent performance in the task - but are still making more effort to inhibit their impulses.

### Limitations and Future Directions

Considering this was a secondary analysis of a modestly sized sample, it is crucial to replicate these findings with a larger sample. Moreover, study variables should be analyzed in a more gender-balanced context, as studies have found sex differences impact social functioning in those in recovery (4). Beyond the sample limitations, our study is limited by the complexity of studying social functioning. Due to its relative neglect in research, there are considerable challenges with operationalizing the concept, finding a standardized measure, and establishing an effective methodology for its study. Future research is needed in which participants are recruited based on their social functioning levels while implementing a standardized measure and an experimental approach to evaluate this variable comprehensively. While validated measures were used, the impact of social desirability and self-report items should still be considered. Finally, despite being a more trauma-informed and community-engaged option for neuroimaging studies, fNIRS restricted our analysis solely to the PFC. Therefore, more investigation is needed, taking advantage of other technologies to analyze these relationships in different brain areas. Despite these limitations, this study is, to our knowledge, the first of its kind to uncover impulsivity and childhood trauma as potential variables underlying the social challenges faced by patients in recovery from OUD.

### Clinical Implications

Developing and maintaining relationships is a critical ingredient for the recovery process (77, 29). Approaches that take advantage of interpersonal relationships, such as peer recovery support (78), have shown great promise in SUD recovery. However, some patients face more social functioning challenges than others, which possibly hampers their recovery process. This study aims to acknowledge and identify areas for potential intervention that could aid in the recovery journey. Our results reveal that, beyond self-reported impulsivity, these individuals have differential connectivity in their brains, which may help explain the social challenges they are experiencing. Understanding the neural differences in those with and without social functioning challenges can help us destigmatize patients in recovery.

Additionally, as underscored by Van Reekum et al. (2020), there is an evident gap in addressing the social needs of patients with OUD during recovery (4). Impulsivity, along with corresponding neural alterations, may, hence, represent a new treatment target. Considering the impact of impulsivity on social impairment varies depending on the history of childhood trauma, it is crucial to implement a trauma-informed, holistic, transdiagnostic approach to OUD recovery. This was particularly true when looking beyond the impact of mental health symptoms, which highlights the importance of, in addition to mental health evaluations, implementing trauma history screening before, and throughout the development of the recovery care plan. This could help generate more personalized and productive strategies to support the social functioning needs of those in recovery.

## Conclusion

Social impairment is a hallmark of OUD, and it can severely impact recovery. This study revealed how social challenges are related to impulsivity and that experiencing childhood trauma can further exacerbate this relationship. Although a notable outcome of OUD, depression symptoms can confound relationships that exist beyond its burden, making it crucial for other variables (such as trauma and impulsivity) to be evaluated and accounted for. By understanding what lies behind the social challenges patients face in recovery, we can implement novel targets that maximize social adjustment during and after recovery.

## Figures and Tables

**Table 1:** Demographics, mental health, and substance use history.

Total Sample	Overall (n = 35)	SI-Y (n = 16)	SI-N(n = 19)	Stats
Age	46.5	45.3	47.6	t = .76 (p = 0.45)
Sex At Birth (% Female)	80%	100%	63%	χ^2^ = 74 (p = 0.007)**
Race (% Black) (n = 34)	56%	33%	72%	χ^2^ = 4.4 (p = 0.04) *
Hispanic Y/N (n = 34)	6%	6%	6%	χ^2^ = .01 (p = 0.73)
Education (self) (n = 34)	15%	13%	17%	χ^2^ = 1.83 (p = 0.4)
Did not finish HS	41%	31%	50%	
HS or GED	44%	56%	33%	
Some college or more				
Education (mother) (n = 34)	21%	6%	33%	χ^2^ = 4.7 (p = 0.1)
Did not finish HS	41%	56%	28%	
HS or GED	38%	38%	39%	
Some college or more				
Substance Use Disorder (n = 33)	94%	94%	94%	χ^2^ = .00 (p = 0.97)
Drug Use in Past Year (n = 33)				
Opioids	88%	81%	94%	χ^2^ = 1.3 (p = 0.28)
Stimulants	45%	44%	47%	χ^2^ = .04 (p = 0.85)
Cannabis	36%	31%	41%	χ^2^ = .35 (p = 0.55)
Alcohol	45%	56%	36%	χ^2^ = 1.5 (p = 0.23)
Mental Health Disorder (n = 33)	79%	94%	65%	χ^2^ = 4.2 (p = 0.05)
Impulsivity (n = 35)	73.2	80.2	7.3	t = 3.3 (p = 0.002)**
Depression (n = 35)	15.8	19.3	12.9	t = 2.7 (p = 0.01)**
Adverse Childhood Experiences (n = 35)	5.9	7.3	4.7	t = 2.1 (p = 0.04)*

** p < 0.01,

* p < 0.05

**Table 2:** The main effect of Social Impairment for different optodes.

Optode	F-value	Num DF	Denum DF	P-value
1	7.89	1	100.8	0.006**
2	1.79	1	99.7	.184
3	0.22	1	91.7	.642
4	4.20	1	99.1	.043*
5	1.03	1	81.5	.314
6	1.83	1	99.7	.179
7	0.53	1	87.8	.468
8	2.70	1	66.7	.105
9	2.64	1	85.2	.108
10	9.06	1	75.8	.004**
11	2.15	1	88.2	.146
12	.000	1	93.0	.997
13	2.16	1	97.5	.145
14	3.46	1	82.5	.067
15	7.56	1	95.6	.007**
16	7.33	1	88.1	.008**

* Significant (p <. 05);

** Significant (p<.01);

## Data Availability

The datasets used and/or analyzed during the current study are available from the corresponding author on reasonable request.
